# Reproductive genetics and health

**DOI:** 10.1515/medgen-2024-2036

**Published:** 2024-09-06

**Authors:** Margot J. Wyrwoll, Johanna Steingröver

**Affiliations:** Centre for Regenerative Medicine University of Edinburgh 5 Little France Drive EH16 4UU Edinburgh United Kingdom; Centre of Medical Genetics University and University Hospital of Münster Vesaliusweg 12–14 48149 Münster Germany

**Keywords:** reproduction, infertility, health, genetics, offspring

## Abstract

For those affected, infertility is linked to impaired overall health and reduced life expectancy. In particular, infertile individuals bear an increased risk for cardiovascular disease (CVD) and different types of cancer, partially due to lifestyle differences and to genetic alterations that cause both infertility and an increased cancer risk. Genetic variants causing an increased CVD risk are more commonly found in infertile individuals, but their link to infertility remains unclear. Offspring of infertile couples, conceived via medically assisted reproduction, are as likely as their parents to exhibit or develop adiposity, hormonal alterations such as insulin resistance, and infertility. The effects on health of subsequent generations are completely unclear.

## Introduction

Reproductive genetics has long been a niche topic and may still be today. Although some may deem infertility an inconvenience rather than a disease and do not consider infertility an immediate threat to life, evidence is accumulating that fertility is a “canary in the coal mine” for health and life expectancy outcomes. This might be due to differences in lifestyle between fertile and infertile people, but it may also be due to biological mechanisms.

Considerations may also exist for the offspring of infertile couples who have children through medically assisted reproduction (MAR). Generally, MAR consists of three different techniques: intrauterine insemination (IUI), *in vitro* fertilization (IVF) and intracytoplasmic sperm injection (ICSI). IUI involves inserting sperm directly into a woman’s uterus, whereas IVF refers to incubating the oocyte with sperm *in vitro*, after which the resulting embryo is transferred into the uterus. ICSI is used in case of male factor infertility and involves injecting one immobilized sperm directly into the oocyte. Here, we provide an overview of health-related issues to consider among infertile couples and their offspring.

## Overall health of infertile men

Infertility may manifest independently or as part of a broader syndrome. Notably, a growing focus is on the syndromic manifestation, which is increasingly being discussed as an early biomarker for associated diseases. Multiple studies have shown that impaired fertility constitutes a risk for cancer in both males and females. Men face an elevated risk of developing testicular and prostate cancer, melanoma, bladder cancer, thyroid cancer, lymphoma and leukaemia. This increased risk extends to first- and second-degree family members, implying a shared predisposition [17]. Moreover, infertile males have an overall increased mortality [21], and this risk increases with the severity of spermatogenic failure and the degree to which semen quality is impaired [2]. Additionally, over 50 % of men experiencing infertility have at least one chronic disease or significant general health condition. Besides cancer, the range of associated diseases is extensive, encompassing a higher likelihood of ischemic heart diseases, diabetes mellitus and elevated blood pressure. Additionally, male infertility might be associated with autoimmune disorders and general immunological dysregulations. While the precise mechanisms underlying these correlations observed in epidemiological analyses remain elusive, the hypothesis of pleiotropic genetic risk factors is appealing.

## Overall health of infertile women

Several of these aspects have also been studied in women. Findings show a correlation between female infertility, predominantly associated with polycystic ovary syndrome, and an increased susceptibility to hypertension after age 45, hypercholesterolemia across all age groups and the onset of diabetes mellitus before age 45 [22]. Polycystic ovary syndrome is also associated with an elevated risk of insulin resistance, hyperinsulinemia, dyslipidaemia, and an elevated risk of prothrombotic state, leading to an increased incidence of conditions such as fatty liver disease, subclinical atherosclerosis and endometrial hyperplasia [29]. Menstrual irregularity in women is concomitant with infertility and indicative of an elevated risk for cardiovascular disease (CVD) later in life. Overall, women experiencing subfertility for more than 5 years have a 20 % increased risk of CVD compared to fertile women, and those with a normal body mass index exhibit the highest increase in CVD risk. Early miscarriages may present an overlooked cause of infertility, potentially attributable to thrombophilia. Hypercoagulable states are associated with elevated CVD risk and, inter alia, implied in pregnancy-related complications like preeclampsia. Subfertility and CVD share undetected hypothyroidism as a common underlying possible cause and latent risk factor. Research also suggests that the increased CVD risk might be due to heightened psychological stress, which can manifest in depression or anxiety, and is frequently found in infertile individuals. Another study has confirmed the connection between nulliparity and an elevated risk of overall mortality (combined relative risk of 1.19 for all-cause mortality in nulliparous women), primarily attributed to a higher susceptibility to CVD-related mortality [30]. This increased risk may arise from fluctuations in reproductive hormone levels and hormone replacement therapy, and conditions leading to infertility could be the underlying cause of hormonal disruptions. Diminished ovarian reserve, indicated by high follicle stimulating hormone (FSH), low anti-mullerian hormone (AMH), low antral follicle count and poor response to fertility treatment, is on the spectrum of ovarian dysfunctions. These constellations pose a risk for premature ovarian insufficiency (POI), resulting in an increased susceptibility to infertility, osteoporosis and CVD [37]. A common pathway, however, remains elusive. Another health condition causing infertility and increased CVD risk is endometriosis, which affects 1 in 10 women and causes chronic pelvic pain and infertility. The underlying inflammation and molecular alterations characteristic of endometriosis have been linked to various chronic diseases, including cardiovascular risk factors, endothelial dysfunction, atherosclerosis and an increased risk for certain ovarian cancer subtypes. A monogenic cause of endometriosis is not known. Similarly, uterine fibroids, benign tumours that affect 20–40 % of women during reproductive age, present an increased risk for all-cause mortality as well as mortality from acute coronary syndrome and strokes. While no monogenic causes for uterine fibroids are known, symptomatic fibroids, characterized by heavy bleedings, often result in chronic anaemia, exacerbating the associated health risks [30].

In terms of increased cancer risk, infertile women have been found to have a 22 % increased risk of cancer-related death at any given point [43]. Notably, findings indicate a twofold mortality rate of breast cancer for a group of infertile women with an otherwise low risk of death relative to a fertile control group [36, 43]. Findings also indicate an increased risk of ovarian cancer in women undergoing fertility treatment. This number is comparable to the risk found in an untreated cohort of infertile women, suggesting that the risk may not be due to the treatment itself but rather to the molecular mechanisms of infertility [30]. Further, the altered hormone profiles observed in nulliparous women may also contribute to the development of hormone-sensitive cancers, such as breast, ovarian or endometrial cancer. Consequently, the observed hormone alterations among nulliparous women may also contribute to mortality associated with cancer [30].

The increased risk for certain cancers and CVD that has been identified in infertile individuals is not necessarily only of biological origin but might also be due to lifestyle factors. Previous research has suggested that infertile individuals may have riskier lifestyles, concerning behaviours like alcohol consumption, smoking or unhealthy dietary habits, all of which can contribute to infertility and significantly increase the risk for CVD and cancer [2].

## Shared genetic causes of infertility and further health-related issues

Shared characteristics of infertility and cancer have been extensively examined. Remarkably, 48 % of genes implicated in cancer causation are relevant for the cell survival system. A similar proportion, 44 %, influences cell fate, while the remaining genes affect genome maintenance. All three of these processes are indispensable for the highly regulated processes of oogenesis and spermatogenesis [27].

Pathogenic variants in 25 genes have been found to be associated with both male infertility and cancer in humans. Notably, literature has characterized 19 of these genes as cancer drivers, with most genes being involved in cell survival. These genes are associated with a diverse spectrum of 38 types of malignancies, with breast and pancreatic cancers being the most common ones. Most of the affected men were infertile due to a reduced sperm count or absent sperm (oligo-/azoospermia) [27].

**Figure 1:**
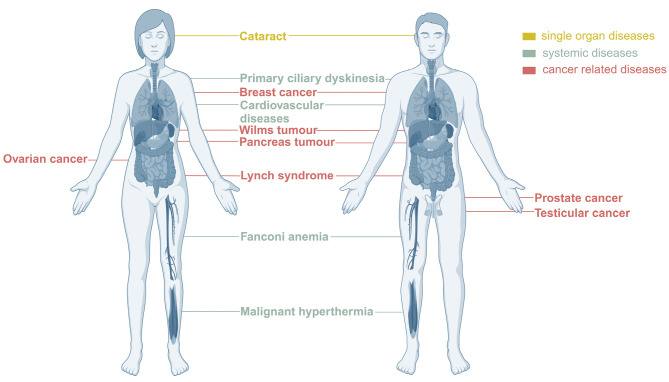
Diseases linked to infertility by shared underlying genetic alterations. Infertility can be linked to single organ diseases like cataract, to systemic diseases like cardiovascular disease and to certain types of cancer.

The *BRCA1* and *BRCA2* genes have emerged as the most commonly affected genes in this context, representing examples of hereditary cancer characterized by defects in DNA repair genes (Table 1, Figure 1). The encoded BRCA1 and BRCA2 proteins preserve genomic integrity and stability by homologous recombination, which facilitates the repair of accidental DNA lesions in somatic cells. Similarly, BRCA1 and BRCA2 are required during gametogenesis to maintain homologous recombination after DNA double-strand breaks are induced physiologically during prophase I of meiosis. It is, thus, straightforward that genetic alterations in one of these genes can hinder DNA repair in somatic as well as germ cells and may predispose individuals to an increased risk of both cancer and impaired gametogenesis [27]. Female infertility increases the likelihood of the presence of pathogenic variants in *BRCA1* or *BRCA2* sevenfold [14]; similarly, infertile men are also prone to pathogenic variants in *BRCA1* or *BRCA2*. For women, pathogenic variants in *BRCA1* or *BRCA2* lead to an elevated risk of early menopause due to reduced ovarian reserve. Patients carrying *BRCA1* variants in particular may have lower AMH levels (*BRCA1* carriers: 1.2±1.1 vs. unaffected individuals: 3.8±2.5 ng/ml) [28]. However, only few women with a pathogenic variant in *BRCA1* or *BRCA2* experience POI, as variants in *BRCA1* and *BRCA2* are not a monogenic cause of infertility. A plausible explanation for this may be that for individuals with a *BRCA*1 or *BRCA2* variant on one allele, the other intact allele is sufficient to maintain DNA repair. Yet, the functionality of this intact allele appears to diminish, starting around age 30–35, potentially leading to clinical implications manifesting after age 37–40 [38].

**Table 1:** Genes linked to infertility and further health conditions.

Genes	Reproductive phenotype	Further health-related issues
**Monogenic gene-disease relationships for infertility**		
*WT1*	Non-obstructive azoospermia, differences in sex development, gonadal dysgenesis	Wilms tumour, Denys-Drash syndrome
*TDRD7*	Non-obstructive azoospermia	Congenital cataract
*CCDC39, CCDC40, CFAP300, DNAAF2, DNAAF3, DNAAF4, DNAH1, DNAH2, DNAH6, DNAH17, DNAI1, DNAI2, DNAJB13, GAS2L2, GAS8, HYDIN, LRRC6, PIH1D3, RSPH3, RSPH4A, SPAG1, SPEF2, ZMYND10*	Asthenoteratozoospermia	Primary ciliary dyskinesia
*FANCA, FANCM, MCM8, SETX, XRCC2, XYCC2*	Non-obstructive azoospermia, premature ovarian insufficiency	Fanconi anaemia
*MCM8, MCM9*	Primary amenorrhea, premature ovarian insufficiency, non-obstructive azoospermia	Diverse congenital malformations, delayed puberty, mental retardation, hearing loss, hypothyroidism, epilepsy, diverse cancer types
**Associations with infertility**		
*BRCA1, BRCA2*	Male/Female infertility	Breast cancer, ovarian cancer, prostate cancer, pancreatic cancer
*MLH1, MLH3*	Non-obstructive azoospermia in male mice with biallelic null alleles; diminished ovarian reserve in female mice with biallelic null alleles; aneuploidy-induced embryo loss in offspring of mice with biallelic missense variants	Lynch syndrome
*KCNQ1, LMNA, MYBPC3, MYH7, MYH11, PKP2, SCN5A, TNNI3*	Male/Female infertility	Cardiovascular disease
*RYR1*	Male/Female infertility	Malignant hyperthermia

Pathogenic variants in the genes *FANCM*, *FANCA*, *XRCC2*, *MCM8*, *MCM9* and *SETX* have been reported to be causal for both isolated and syndromic infertility. Men with pathogenic variants in one of these genes commonly have non-obstructive azoospermia (NOA) [20]. Women harbouring variants in these genes exhibit a spectrum ranging from POI to gonadal dysfunction, depending on the presence of one or two affected alleles [12]. All these genes assume critical functions in DNA repair mechanisms, serving as guardians of genome stability in somatic and germ cells. The genes *FANCM*, *FANCA* and *XRCC2* are also directly involved in the Fanconi anaemia pathway, as biallelic variants in these genes can cause Fanconi anaemia. Infertility is a common feature of Fanconi anaemia, with affected individuals having NOA or POI [12, 20].

Pathogenic variants in *MLH1*, a component of the mismatch repair pathway, cause Lynch syndrome and have been identified as a risk factor for oligo-/azoospermia in men [39]. Moreover, findings indicate an association between biallelic missense variants in *MLH1* as well as* MLH3* and aneuploidy in offspring, pregnancy loss and premature reproductive aging in female mice [32]. Similarly, variants in *WT1* are mainly known as the cause of Wilms tumour. As a feature of Denys-Drash syndrome, variants in exon 8 and 9 of *WT1* can cause congenital nephropathy and differences in sex development (DSD) or complete gonadal dysgenesis. However, missense variants in this gene have also been described as the reason for isolated male infertility due to spermatogenic failure.

An intriguing example of genetic variants affecting multiple cellular processes across different developmental stages are pathogenic variants in *TDRD7*. Loss-of-function variants in *TDRD7* cause a syndrome characterized by congenital cataracts in both sexes and non-obstructive azoospermia in males. During development of the lens, TDRD7 is required for autophagosome maturation, which is crucial for lens transparency by facilitating the removal of damaged proteins. TDRD7 is also required in adult males for formation of the acrosome during spermiogenesis.

Another syndromic condition involving infertility is primary ciliary dyskinesia (PCD). PCD is characterized by a malfunction of motile cilia located in the nose, ears and lungs, causing impaired clearance of the mucus, ultimately leading to chronic respiratory infections. Patients may also have a situs inversus, also called Kartagener syndrome. Diagnosing PCD involves a comprehensive approach, such as sampling the nasal epithelium to evaluate ciliary function and structure. PCD is a heterogeneous disease, as variants in more than 50 genes have been described as causal [19, 31]. Depending on the gene affected, male infertility is also possible, given that parts of the axoneme in the respiratory cilia are also part of the sperm flagellum. These men commonly feature immotile sperm or sperm with reduced motility (asthenozoospermia). Moreover, the morphology of the sperm can be altered, with sperm having short or bent flagella (teratozoospermia). However, some components of the axoneme are only found in sperm and not in respiratory cilia. If these are affected by pathogenic variants, the resulting phenotype is limited to infertility due to asthenoteratozoospermia, which is commonly referred to as multiple morphological abnormalities of the sperm flagellum (MMAF). In some instances, PCD is not diagnosed until infertility evaluation. Women with PCD may also be infertile due to a compromised function of the cilia in the fallopian tube [41]. However, female and male infertility in PCD patients can be overcome by MAR.

In addition to genetic variants, some researchers have suggested epigenetic alterations as a driver of the association between infertility and cancer, which may serve as a common underlying factor for both conditions. This assumption arose from the observation of similar impaired pathways, alterations in imprinted loci and histone modifications for both cancer and infertility patients. One study suggested a “two-hit” hypothesis to explain how the epigenetic alterations are induced, whereby one epimutation might predispose individuals to infertility, while another one might increase their susceptibility to cancer, mirroring the well-established paradigm observed in the development of cancer [18].

Although a few studies have found an association between CVD and infertility, the genetic link is far from clear. In different studies, genetic variants in the genes* KCNQ1, MYBPC3, MYH7, MYH11, LMNA, PKP2, SCN5A* and *TNNI3* have been found to appear more frequently in cohorts of infertile probands than in healthy individuals. While these variants represent well-known causes for cardiomyopathy, arrhythmia and aortic aneurysm, their connection to infertility is still unclear.

## Health of infertile couples’ offspring

Because infertile couples tend to have generally reduced overall health, it seems reasonable to assume that their offspring might also experience more health issues relative to fertile couples’ offspring. Yet, it may be hard to differentiate whether such health issues arise from inheriting medical predispositions or from the process of MAR. According to a systematic review considering 21 studies from the years 2000 to 2016, ICSI children have a 7.1 % overall risk for congenital malformation, whereas that for naturally conceived children is 4.0 % [23]. Unfortunately, only few studies have focused on both congenital health issues and the long-term health of children conceived by ICSI. According to a systematic review, compared to spontaneously conceived children, ICSI children have similar neurodevelopment, growth, vision and hearing [9]. Still, the relative risk for autism in children conceived by MAR seems to be increased by a factor of 1.35, as revealed by another meta-analysis [25], but this might also be due to increased paternal age. Furthermore, ICSI children have reduced general physical health, resulting in higher rates of surgical interventions, hospitalization and medical therapy, as found in some studies (Table 2) [7]. Nevertheless, all included studies in this systematic review show weaknesses and no profound effects [9], so further studies are necessary to clarify these associations. Another increasingly recognized concern is that children conceived by ICSI as well as other MAR methods might have increased cardiovascular and metabolic risks, as they tend to show higher rates of adiposity [4], hormonal alterations including increased cortisol levels [5], and increased risk for insulin resistance [10, 11]. Researchers have hypothesized that the time around conception represents a critical period for epigenetic reprogramming, with alterations in this process causing long-term health effects such as metabolic disorders [40]. Another issue for ICSI children is impaired reproductive health, resulting in reduced semen quality in young men who are conceived by ICSI [3]. Most fathers of these men were only able to conceive with the help of MAR [3], such that this condition may be inherited. However, data on this topic are still sparse, which precludes any general conclusions.

A recent systematic review and meta-analysis has tried to overcome the issue of sparse data and methodologically weak studies by analysing data from animal studies, which do not rely on subjective reports of, e. g., parents. This meta-analysis revealed that the use of IVF/ICSI is associated with a longer length of gestation and higher birth weights across different species, including cattle, mice, sheep, horses and primates [1]. Although data on CVD and insulin resistance in animals have been inconclusive, these conditions may have the same underlying mechanisms as increased gestational length and birth weights. Although more data are available when analysing animal studies, the data from single studies included in the analysis exhibit heterogeneity. Moreover, MAR techniques used in animals differ from those used in humans, thus hampering direct comparisons.

Studies have repetitively shown that mice conceived by ICSI exhibit behavioural abnormalities. The phenotypes of affected mice in these studies are particularly characterized by increased anxiety and a disorder resembling depression. A recent study has investigated the abnormal phenotype of the offspring of these mice, labelled as the second generation [24]. These offspring were conceived by IVF from the sperm of the first-generation mice, which were conceived by ICSI and appeared physically normal besides the behavioural changes. Surprisingly, the incidence of hydrocephalus in the second generation was 137.9-fold higher relative to wild-type mice [24]. Additionally, anophthalmia, small eyes and skull defects were observed more frequently in mice of the second generation. No evidence has suggested that these abnormalities are attributable to imprinting defects; hence, these findings stand in contrast to the widely accepted hypothesis that ICSI-induced health issues may be linked to imprinting defects.

**Table 2:** Studies reporting on general physical health in ICSI- and spontaneously conceived offspring. Table taken from Catford et al., 2018 [9] with permission from John Wiley and Sons

Authors, year location	Study design	Study groups and sample size	Outcome measures	Age	Plurality	Gestation	Key results	Quality assessment using NOS	
								Total score	AHRQ standard
Belva *et al.*	Prospective	150 ICSI	Parent questionnaire;	8 years	S	≥32 weeks	Median DBP and SBP higher in ICSI	6	Fair
(2007), Belgium	cohort	147 SC	physical, and neurological				vs. SC group (SBP 100 vs.		
			examination including				95 mmHg, *p* = 0.0007; DBP 60 vs.		
			visual acuity and				55 mmHg, *p* < 0.0001);		
			audiometry; Tanner staging^a^				diadochokinesis (coordination) performed better by SC group		
							(*p* < 0.0001); fingertip-touching		
							(coordination) performed better by		
							ICSI group (*p* = 0.0013); no		
							differences in general health,		
							pubertal staging, neurological		
							development or need for remedial		
							therapy, surgery, or hospitalizations		
Bonduelle *et al.*	Retrospective	300 ICSI	Parent questionnaire;	5 years	S	≥32 weeks	More surgical interventions in ICSI vs.	6	Fair
(2004), Belgium,	cohort	266 SC	physical and neurological				SC children (23 vs. 16.5 %,		
Sweden, USA			examination including				*p* = 0.019)—mainly higher rate		
			visual acuity and audiometry^a^				minor ear problems (tympanic drains and adenoidectomy);		
							physiotherapy, speech, orthoptic,		
							dietary and psychological therapy		
							required more in ICSI vs. SC group		
							(13.6 vs. 6.2 %, *p* = 0.012); no		
							difference in growth, chronic		
							diseases & overall health		
Bonduelle *et al.*	Cross-sectional	540 ICSI	Parent interview; physical	5 years	S	≥32 weeks	ICSI and IVF groups more likely to	6	Fair
(2005), Belgium,	cohort	437 IVF	examination including				have significant childhood illness		
UK, Denmark, Sweden, Greece	(same cohort as Barnes	538 SC	visual acuity and audiometry^a^				(74 % ICSI, 77 % IVF, 57 % SC; *p* < 0.001), need surgery (24 % ICSI,		
	*et al.*, 2004)						22 % IVF, 14 % SC; *p* < 0.001) esp.		
							genitourinary surgery (5 % ICSI, 3 %		
							IVF, 1 % SC; *p* = 0.005), require		
							medical therapy (11 % ICSI, 9 % IVF,		
							5 % SC; *p* < 0.001), and be		
							hospitalized (31 % ICSI, 28 % IVF,		
							20 % SC; *p* < 0.001) compared to		
							SC group		
Kettner *et al.*	Retrospective	2389 ICSI	Diagnosis of T1DM by	13–14 years	S	Any	No association between IVF/ICSI or	8	Good
(2016), Denmark	cohort	5195 IVF	insulin prescription via				OI/IUI and childhood T1DM; OI or		
		14,985 OI or IUI	Danish National				IUI with FSH association with		
		541,641 SC	Prescription Registry				increased risk T1DM (HR 3.22, 95 %		
							CI 1.20–8.64); no association		
							between ICSI and T1DM (aHR 0.99,		
							95 % CI 0.49–1.98); no association		
							between IVF and T1DM (aHR 1.05,		
							95 % CI 0.67–1.66)		
Knoester *et al.*	Cross-sectional	81 ICSI vs. 81 IVF	Parent questionnaire;	5–8 years	S	Any	No difference in general health,	5	Poor
(2008a,b), Holland	cohort	87 ICSI vs. 85 SC	physical examination				growth, or hospitalizations between		
			including biometrical data and vision^a^				ICSI and IVF or SC groups; higher rate of physical therapy in IVF vs.		
							ICSI group (OR 2.6, 95 % CI 1.0–		
							6.6)		
Ludwig *et al.*	Prospective and	276 ICSI	Parent interview and	4–6 years	S	≥37 weeks	Significantly increased risk of	6	Fair
(2009b),	cross-sectional	(prospective)	questionnaire; physical				undescended testes (5.4 vs. 0.7 %,		
Germany	cohorts	273 SC	examination including				*p* = 0.031) and more urogenital		
	(ICSI cohort	(cross-sectional)	visual acuity and				surgeries in ICSI vs. SC boys (19.2		
	same as		audiometry				vs. 8.9 %, *p* = 0.013); more ICSI		
	Ludwig *et al.*,						children hospitalized (37.6 vs.		
	2009a, 2010)						27.2 %, *p* = 0.006) (no specific		
							reason); no differences in incidence		
							of childhood diseases, acute and		
							chronic illnesses, accidents and		
							overall surgeries		

aHR, adjusted hazards ratio; AHRQ, Agency for Health Research and Quality; BW, birth weight; DBP, diastolic blood pressure; GA, gestational age; IUI, intrauterine insemination; NOS, Newcastle–Ottawa Scale; OI, ovulation induction; PR, participation rate; S, singleton birth; SBP, systolic blood pressure; SC, spontaneously conceived; T1DM, type I diabetes mellitus. ^a^Also reported perinatal and/or obstetric outcomes and/or congenital malformations.

It may be difficult to attribute health impairments in offspring to single factors, as several risk factors often accumulate in couples struggling to conceive. The cumulative effect of these factors is even more difficult to estimate. Research has speculated that the increased risks following ICSI are partly due to the hydrolysing enzymes contained in the acrosome of sperm. Unlike during natural conception, these enzymes are incorporated into the oocyte as part of the ICSI procedure [26]. It has further been surmised that the artificial environment during MAR, including freezing of embryos and gametes, using culture media and the delayed insemination, may explain possible epigenetic alterations in the embryo [25]. It has also been suggested that the increased atmospheric oxygen levels for MAR conditions compared to *in vivo* conditions lead to DNA damage via oxidative stress. Yet, it seems that the detrimental effect of oxidative stress is exclusively present from the cleavage stage on before embryonic genome activation [16].

On top of technical factors increasing the health risks for offspring conceived via ICSI, the transfer of multiple embryos, constitutes an additional risk for the offspring and the mother therefore single embryo transfer has long been recommended [15]. Additionally, couples requiring medical assistance to conceive are commonly older than couples conceiving naturally. Women in Germany are, on average, 31.6 years old when they give birth to any child [8], whereas the average woman’s age in Germany at birth of the first child being 30.1 years [35]. In contrast, based on data from 2022, the average age of women undergoing IVF/ICSI is 35.8 years [13]. This higher maternal age favours chromosomal disorders, resulting in an increased rate of such disorders as well as miscarriages. Of note, the risk for chromosomal anomalies seems to be increased in ICSI children independent of maternal age. A meta-analysis from 2021 including cohort studies with systematic cytogenetic testing found a prevalence of chromosomal abnormalities of 1.3–4.3 % in children conceived by ICSI, compared to a prevalence of 0–0.9 % in children conceived naturally, after adjusting for maternal age [6]. Unfortunately, data on the chromosomal abnormalities in children conceived by the less invasive IVF are not available.

Just as the maternal age is higher in couples undergoing IVF/ICSI, so is the paternal age. The average age of men receiving MAR in Germany is 38.6 years [13], compared to 34.6 years in the total population [34]. Increased paternal age is associated with *de novo* mutations, but IVF and ICSI do not seem to additionally increase the risk of *de novo* mutations in children [33]. In children born to young fathers (<35 years of age), 71 *de novo* mutations have been found on average, whereas in children born to older fathers (>45 years of age), 94 *de novo* mutations have been found on average, independent of the mode of conception [33]. 

Lately, ICSI has been increasingly used in couples with non-severe male factor infertility. In light of this development, a very recent study has advised against ICSI in this context, as ICSI does not improve the live birth rate but represents an extremely invasive procedure [42]. However, another very recent study did not detect any differences in rates of miscarriages and congenital malformations between IVF and ICSI children, with rates for congenital malformations in live born children being 5.4 % and 5.8 %, respectively [44]. Nevertheless, the use of IVF is still preferable to ICSI in case of non-severe male factor infertility [42].

## Conclusion

Several studies have revealed that infertile individuals are at an increased risk for multiple diseases including different types of cancer and CVD. In line with this, infertile individuals tend to have reduced life expectancy. While in most cases the mechanism is not clear, the reasons for increased cancer and CVD exposure in the case of infertility may not exclusively be biological. Still, further research is required to elucidate these mechanisms, especially to decipher the link between infertility and CVD and to further investigate the health of offspring. As the first generation of individuals conceived by MAR is now of reproductive age, we will soon learn more about underlying effects for subsequent generations in humans. The field is evolving rapidly, and MAR has become globally available, enabling large-scale studies. Considering the potential transgenerational impacts, studying reproductive health is of paramount societal interest and represents a top-tier medical objective.
